# Anti-Inflammatory Effect of Excretion-Secretion Products of *Clinostomum marginatum* (Digenea: Clinostomidae) and Its Effect over the Viability and Antioxidative Activity of a Mix of *Lactobacillus* and/or *Bifidobacterium*

**DOI:** 10.3390/microorganisms14020354

**Published:** 2026-02-03

**Authors:** María Angélica Gutiérrez-Nava, Raquel González-Vázquez, Miguel Ángel Mosqueda-Cabrera, Daniela Reyna-González, Felipe Mendoza-Pérez, Eduardo Zúñiga-León, Leovigildo Mateos-Sánchez, Pedro A. Reyes-Castillo, Rosa González-Vázquez, María Guadalupe Córdova-Espinoza, Alejandro Escamilla-Gutiérrez, Luis Alberto Reyes-Nava, Lino Mayorga-Reyes, Ana Laura Esquivel-Campos

**Affiliations:** 1 Laboratorio de Ecologia Microbiana, Departamento de Sistemas Biologicos, Universidad Autonoma Metropolitana Unidad Xochimilco, Calzada del Hueso 1100, Colonia Villa Quietud, Alcaldia Coyoacan, Ciudad de México 04960, Mexico; 2 Laboratorio de Biotecnologia, Departamento de Sistemas Biológicos, SECIHTI-Universidad Autónoma Metropolitana Unidad Xochimilco, Calzada del Hueso 1100, Colonia Villa Quietud, Alcaldia Coyoacan, Ciudad de México 04960, Mexico; rgonzalezv@correo.xoc.uam.mx; 3 Departamento El Hombre y su Ambiente, Universidad Autónoma Metropolitana Unidad Xochimilco, Calzada del Hueso 1100, Colonia Villa Quietud, Alcaldia Coyoacan, Ciudad de México 04960, Mexico; zitzitl@correo.xoc.uam.mx; 4 Laboratorio de Biotecnologia, Departamento de Sistemas Biológicos, Universidad Autonoma Metropolitana Unidad Xochimilco, Calzada del Hueso 1100, Colonia Villa Quietud, Ciudad de México 04960, Mexico; 2203059038@alumnos.xoc.uam.mx (D.R.-G.); fmendoza@correo.xoc.uam.mx (F.M.-P.); jezuniga@correo.xoc.uam.mx (E.Z.-L.); 5 Unidad de Cuidados Intensivos Neonatales, UMAE Hospital de Gineco Obstetricia No. 4 Luis Castelazo Ayala, Instituto Mexicano del Seguro Social (IMSS), Ciudad de México 06720, Mexico; lmateos95@yahoo.com.mx; 6 Unidad Médica de Alta Especialidad, Hospital de Oncología, Centro Médico Nacional Siglo XXI, Instituto Mexicano del Seguro Social (IMSS), Ciudad de México 06720, Mexico; reyes.castillo.pedroa@gmail.com; 7 Laboratorio de Bacteriologia Medica, Departamento de Microbiologia, Escuela Nacional de Ciencias Biológicas, Instituto Politecnico Nacional, Prolongacion de Carpio y Plan de Ayala S/N, Col. Casco de Santo Tomas, Alcaldia Miguel Hidalgo, Ciudad de México 11340, Mexico; rosagonzvazq@yahoo.com.mx (R.G.-V.); mixtlipp@yahoo.com.mx (M.G.C.-E.); alescamillag@hotmail.com (A.E.-G.); 8 Unidad Medica de Alta Especialidad, Hospital de Especialidades, “Dr. Antonio Fraga Mouret”, Centro Medico Nacional La Raza, Instituto Mexicano del Seguro Social (IMSS), Ciudad de México 02990, Mexico; 9 Laboratorio de Inmunología, Escuela Militar de Graduados de Sanidad, Secretaria de la Defensa Nacional, Ciudad de México 11200, Mexico; 10 Unidad Medica de Alta Especialidad, Laboratorio de Microbiologia, Hospital General “Dr. Gaudencio González Garza”, Centro Medico Nacional La Raza, Instituto Mexicano del Seguro Social, Ciudad de México 02990, Mexico; 11 Departamento de Ciencias de la Naturaleza, Universidad de Guadalajara, Centro Universitario del Sur, Av. Enrique Arreola Silva No. 883, Colonia Centro, Ciudad Guzman 49000, Mexico; luis.reyes@cusur.udg.mx

**Keywords:** *Clinostomum marginatum*, inflammation, microbiota, anti-inflammatory, antioxidant, *Lactobacillus*, *Bifidobacterium*

## Abstract

The trematode *Clinostomum marginatum*, secretes excretory-secretory products (ESPs) which have the potential to increase the viability and antioxidant activity of probiotic strains. The aim of this study was to identify the ESP profile of *C. marginatum* and to evaluate its anti-inflammatory activity in RAW 264.7 macrophages, as well as its effect on the viability and antioxidant activity of a consortium of bacteria comprising *Lactobacillus* and/or *Bifidobacterium*. *C. marginatum* was maintained in RPMI-1640 medium for ESP collection. Anti-inflammatory activity was assessed in LPS-stimulated RAW264.7 cells treated with 800 µg/mL of ESPs, measuring cell viability, nitric oxide production, and the relative expression of pro-inflammatory cytokines (IL-6, TNF-α, INF-γ) and the COX-2 gene by qPCR. The influence of ESPs (800–1600 µg/mL) on probiotic viability and antioxidant activity was determined using MTT, DPPH, hydroxyl, and superoxide radical scavenging assays. *C. marginatum* showed 74% survival in vitro, and SDS-PAGE analysis revealed three major protein bands in the ESPs (47, 54, and 58 kDa). ESP treatment significantly reduced nitric oxide and the mRNA expression of pro-inflammatory markers in LPS-activated macrophages. ESPs supplemented at 1200 µg/mL optimized the growth kinetics of *Lactobacillus* (specific growth rate *μ_L_* = 1.12 h^−1^, doubling time *t_d_* = 0.62 h) and *Bifidobacterium* (*μ_B_* = 1.09 h^−1^, *t_d_* = 0.63 h) compared to control conditions. In conclusion, ESPs from *C. marginatum* exhibited significant anti-inflammatory and antioxidant effects while enhancing bacterial viability, which positions them as promising candidates for biotherapeutics agents in the management of inflammatory control and gut microbiota modulation.

## 1. Introduction

Inflammation is a fundamental innate immune response elicited by pathogens, cellular debris, or irritants. However, a persistent or dysregulated response can progress to chronic or systemic inflammatory disorders [1]. Chronic degenerative diseases (CDDs) encompass a diverse group of non-infectious, slowly progressing, and long-lasting conditions, including obesity, rheumatoid arthritis, diabetes, respiratory and cardiovascular diseases, osteoporosis, sarcopenia, renal failure, inflammatory bowel diseases, Huntington’s disease, and various types of cancer [2]. These pathologies are frequently associated with a state of chronic, low-grade systemic inflammation termed “inflammaging”, a condition linked to aging and characterized by the dysregulation of multiple cellular and molecular pathways [3]. Globally, CDDs are the leading cause of disability and mortality, affecting more than 30% of the population and accounting for over 70% of public health expenditures [2,4].

Macrophages (MΦ) are pivotal mediators of the inflammatory response due to their ability to secrete pro-inflammatory cytokines and produce inducible nitric oxide synthase (iNOS) expression [5]. Nitric oxide (NO) and reactive oxygen species (ROS) play central roles in modulating immune responses and activating inflammatory signaling pathways. Pro-inflammatory cytokines such as interleukins-1β (IL-1β), interleukin-6 (IL-6), interleukin-12 (IL-12), interleukin-23 (IL-23), and tumor necrosis factor alpha (TNF-α), along with bacterial lipopolysaccharide (LPS), are potent inducers of iNOS expression [6,7,8]. Similarly, cyclooxygenase-2 (COX-2) is an inducible enzyme responsible for the synthesis of prostaglandins (PGs) and the generation of ROS. Although the pharmacological inhibition of COX-2 by nonsteroidal anti-inflammatory drugs (NSAIDs) reduces ROS levels and subsequently suppresses the production of key inflammatory mediators (PGs, NO, IL-6, and TNF-α), prolonged NSAID use is often associated with significant adverse gastrointestinal and renal effects. This underscores the imperative need for the development of safer and more effective anti-inflammatory alternatives [9].

Ethnobotany and ethnopharmacology have historically contributed to the identification of bioactive compounds capable of modulating inflammatory processes [10]. Nevertheless, given that these approaches often prove insufficient, there is a critical need for novel strategies to prevent and manage CDD-associated inflammation. In this context, the “hygiene hypothesis” proposes that reduced exposure to helminths during childhood increases the susceptibility to allergic, inflammatory, and metabolic disorders in adulthood [11]. Indeed, helminth infections, characterized by the release of ESPs into the host environment, have been shown to attenuate allergic, autoimmune, and inflammatory responses, primarily through the activation of anti-inflammatory signaling pathways [12,13,14]. Although helminths infect nearly one-fourth of the global population, these infections are rarely lethal, reflecting a long-term evolutionary co-adaptation between parasites and their hosts [12,15]. Recent evidence indicates that helminths further modulate gut microbiota composition and diversity [16], thereby influencing susceptibility to conditions such as asthma, colitis, viral infections, and metabolic disorders [17]. Notably, Marsh et al. (2024) [18] positioned helminths as ecological drivers that shape host-microbiota interactions and overall health, offering a potential biological alternative to conventional NSAIDs.

In this context, *Clinostomum marginatum* (Digenea: Clinostomidae) is a digenetic trematode that parasitizes a variety of freshwater and estuarine fish species, including *Dormitator maculatus*. The latter, known locally as “chucumite”, is traditionally consumed in several coastal regions of Mexico. *D. maculatus* undergoes reproductive migrations within estuarine systems; in the Alvarado Lagoon, Veracruz, local communities capture females to extract and consume their gonads. This traditional practice is believed to promote nutritional health—providing essential proteins and lipids—rather than serving merely as a seasonal delicacy [19]. Such traditional consumption underscores the importance of characterizing associated parasites, which may represent an unexplored source of bioactive molecules. Given the increasing interest in helminth-derived molecules with immunomodulatory potential, the study of ESPs [20] from *C. marginatum* isolated from *D. maculatus* provides a unique opportunity to explore their putative anti-inflammatory and microbiota-modulating properties. Therefore, the aim of this study was to identify excretory-secretory products from the parasite *C. marginatum* and to evaluate their anti-inflammatory activity using an in vitro macrophage model. Additionally, the effect of these ESPs was determined on the viability and antioxidant activity of a probiotic consortium comprising *Lactobacillus* and/or *Bifidobacterium*.

## 2. Materials and Methods

### 2.1. Isolation and Characterization of Helminth-Derived Products

#### 2.1.1. Helminths Collection

Metacercariae of *C. marginatum* were isolated from the coelomic cavity, liver, and gonads of *D. maculatus* species captured in temporary water bodies within the Papaloapan River basin. Fish were obtained from commercial vendors in Tlacotalpan Veracruz, México.

To release helminths from host tissue, the organs were subjected to enzymatic digestion using a solution containing 6 g pepsin, and 7 mL hydrochloric acid in 1 L of distilled water, at 37 °C for 30 min. The recovered metacercariae were subsequently washed four times with 0.6% saline solution supplemented with 100 U/mL of penicillin-streptomycin (Invitrogen, Carlsbad, CA, USA) [21].

#### 2.1.2. Recovery and Purification of ESPs

Metacercariae was initially cultured in RPMI 1640 medium supplemented with 10% fetal bovine serum (Gibco, Waltham, MA, USA), penicillin-streptomycin (100 U/mL), and 2 mM glutamine (Invitrogen USA) using 6-well plates. Cultures were maintained in a humidified atmosphere with 5% CO_2_ for two weeks for in vitro adaptation. Following this period, the medium was replaced with serum-free RPMI 1640 containing penicillin-streptomycin (100 U/mL), and 1 mM sodium pyruvate for a duration of two months [22,23]. During this phase, 1 mL of medium was collected at seven-day intervals (weekly) resulting in eight collected samples (batches) and replaced with fresh medium.

The collected ESPs were quantified using the Lowry method. To ensure batch reproducibility, protein concentration was measured individually in each of the eight batches obtained over the two-month period. No significant variations in protein yield or electrophoretic patterns were observed between batches, confirming a stable secretory profile during in vitro cultivation. Consequently, the eight batches were pooled to ensure a homogenous mixture for all subsequent biological assays; the results reported herein reflect the average protein concentration of this pool. The qualitative purity of the ESPs was assessed via 10% sodium dodecyl sulfate-polyacrylamide gel electrophoresis (SDS-PAGE) according to the Laemmli method [24,25], specifically to verify the absence of high-molecular-weight degradation products and to confirm the consistency of the protein bands across all batches. ESPs were further purified by dialysis against PBS buffer using a 10 kDa molecular-weight cut-off (MWCO) membrane, lyophilized (−40 °C, 0.200 mBar) for 24 h, and reconstituted in 1 mL of Milli-Q sterile water. Molecular weight determination was performed using protein markers from 25 to 250 kDa [26,27]. Gels were silver-stained with 20 mM AgNO_3_ for 30 min, then the image was developed in a solution of Na_2_CO_3_ (0.28 M) and 0.037% CH_2_O, and the reaction was stopped with 1% CH_3_COOH for 30 min. To accurately estimate the molecular weight of the ESP bands, a standard calibration curve was constructed by plotting the logarithm of the molecular weight of the protein markers against their relative mobility (*Rf*). The molecular weight of the ESP proteins was then calculated by interpolating their *RF* values into the resulting linear regression equation [28].

### 2.2. In Vitro Evaluation of Biological and Molecular Effects

#### 2.2.1. Cell Viability Assay (MTT)

RAW 264.7 murine MΦ were cultured in RPMI-1640 medium supplemented with 10% FBS and penicillin-streptomycin (Invitrogen, USA, 100 U/mL) at 37 °C under 5% CO_2_. Cell viability was assessed using the MTT assay (3-(4,5-dimethyl-2- thiazolyl)-2,5-diphenyl-2H-tetrazolium bromide). Briefly, 4 × 10^4^ cells were seeded in 96-well plates and incubated for 24 h at 37 °C under 5% CO_2_. Cells were then treated with ESPs at concentrations ranging from 25 to 2000 µg/mL. Subsequently, 20 μL of MTT solution (0.5 mg/mL) was added to each well and incubated for 4 h at 37 °C. The solution was removed, and formazan crystals were solubilized in 200 μL of dimethyl sulfoxide. Optical density (OD) was measured at 540–570 nm. Viability is expressed as a percentage relative to control cells (100% viability) [29]. The experiment comprised three independent biological replicates, and each performed in triplicate.

#### 2.2.2. In Vitro Anti-Inflammatory Assay

To evaluate the anti-inflammatory potential, 3 × 10^5^ MΦ were seeded per well and assigned to five groups: (i) Control (untreated MΦ); (ii) MΦ treated with 31 µM of diclofenac; (iii) MΦ stimulated with LPS (1 µg/mL *E. coli* O111:B4); (iv) MΦ treated with 31 µM of diclofenac, followed by the addition of 1 µg/mL LPS after 2 h; (v) MΦ stimulated with 800 µg/mL ESPs, followed by the addition of 1 µg/mL LPS after 2 h of incubation. All cells were incubated for 24 h at 37 °C and 5% CO_2_. 800 µg/mL of ESPs was selected as the maximum non-toxic dose based on viability assays. All ESP dosages were standardized based on protein content determined by the Lowry method. Supernatants were collected for the nitric oxide (NO) quantification, and total RNA was extracted for gene expression analysis [30]. The experiment comprised three independent biological replicates, each performed in a triplicate.

#### 2.2.3. Nitric Oxide Quantification

NO production in the culture supernatants was determined using the Griess reagent (PROMEGA G2930 kit, Promega, Madison, WI, USA) according to the manufacturer’s instructions. A standard curve was generated using nitrite standards (0–100 µM/mL) diluted in culture medium. OD was measured at 540 nm using an automatic plate reader (Bio-Rad Laboratories, Hercules, CA, USA) [30].

#### 2.2.4. Quantitative REAL-TIME PCR Analysis

Total RNA was extracted using the TRIzol reagent (Invitrogen, USA). Concentration and purity were assessed by spectrophotometry at 260/280 nm. One microgram of total RNA was reverse transcribed into complementary DNA (cDNA). qRT-PCR was conducted using SYBR Green^®^ assays and the specific primers listed in Table 1. The expression levels of IL-6, TNF-α, interferon gamma (INF-γ), and COX-2 were normalized to 18S mRNA as an endogenous control. Relative expression was calculated using the ^ΔΔ^Ct method [31].

### 2.3. Probiotic Viability and Antioxidant Activity Assays

#### 2.3.1. Bacterial Strains and Cellular Fractions Preparation

The strains *Lactobacillus rhamnosus* LBUX2302 (Genbank PQ724459), *L. rhamnosus* LBUX2304 (Genbank SV15654237), *Lacticaseibacillus paracasei* LBUX2305 (Genbank PV682648), *L. rhamnosus* LBOX2312 (Genebank SV15654270), *Bifidobacterium longum* LBUX23 (Genbank CP116390) and *Bifidobacterium pseudocatenulatum* JCLA3 (Genbank CP090598) were previously isolated from human gut microbiota and were selected for this study because they showed probiotic potential [37,38]. *L. casei* was previously isolated from a commercial product (Yakult Honsha Co., Ltd., Tokyo, Japan ^®^) [39] and *B. animalis sub lactis* BB12 was obtained from CHR Hansen; both strains were used as control in viability and antioxidant activity assays. *Lactobacillus* strains were cultured in MRS broth (Man, Rogosa, and Sharpe) and *Bifidobacterium* in MRS broth supplemented with 5% cysteine and CO_2_. The cultures were incubated at 37 °C for 24 to 48 h. Bacterial growth was monitored by plate count until reaching the concentration of 1 × 10^8^ CFU/mL before further experimental assays [37,38]. A consortium of *B. longum* LBUX23 and *B. pseudocatenulatum* JCLA3 (considered as B consortium) and a consortium of *L. rhamnosus* LBUX2302, *L. rhamnosus* LBUX2304, *L. paracasei* LBUX2305 and *L. rhamnosus* LBOX2312 (considered as L consortium) were prepared at a final concentration of 1 × 10^8^ CFU/mL each, under the experimental conditions mentioned above. These consortiums were employed in experiments involving intact cells, supernatant, or lysed cell fractions. Control strains were employed at a concentration of 1 × 10^8^ CFU/mL, under the same experimental conditions.

To obtain intact cells and the cell-free supernatant, all cultures were centrifuged at 812× *g* for 5 min. The resulting cell pellets were washed twice with PBS (pH 7.4) and recovered by centrifugation under the same conditions prior to their use in antioxidant assays. Additionally, a fraction of the washed cells was subjected to chemical and mechanical lysis. Briefly, ZymoBIOMICSTM Lysis Solution was added in a 2:1 ratio, and cells were disrupted through five cycles of 1 min homogenization, each interspersed with 3 min of cooling on ice to preserve molecular stability [37,38].

#### 2.3.2. Antioxidant Activity

The scavenging activity against 2,2-diphenyl-1-picrylhydrazyl (DPPH), hydroxyl and superoxide radicals was evaluated for each consortium according to the methodology reported by Reyes-Castillo et al. (2023) [37,38]. Treatments were standardized using a final concentration of 800 µg/mL of ESPs. The experimental groups comprised the *Bifidobacterium* consortium supplemented with ESPs (B + ESPs), the *Lactobacillus* consortium supplemented with ESPs (B + ESPs) and the *Bifidobacterium-Lactobacillus* consortiums supplemented with ESPs (B + L + ESPs). *L. casei* and *B. animalis sub lactis* BB12 were used as control strains [37,38].

##### DPPH Radical Scavenging Activity

The scavenging activity against DPPH radical was determined using a 0.2 mM methanolic solution. Briefly, 1 mL of the DPPH reagent was mixed with 1 mL of either intact cells, cell lysates or cell-free supernatants. Blank samples consisted of a lysis buffer/PBS mixture (1:3 *v*/*v*) and sterile MRS medium to account for background absorbance from each consortium fractions (intact cells, cell-free supernatant, or lysate). Parallel control samples were prepared by mixing 1 mL of each fraction with 1 mL of PBS (pH 7.4). All reaction mixtures, including controls and blanks, were incubated for 30 min at room temperature in the dark. Following incubation, the OD was measured at 517 nm. *L. casei* was employed as a control strain [37,38].

##### Hydroxyl Radical Scavenging Activity

The hydroxyl radical (⸱OH) inhibition capacity was assessed in intact cells, cell lysates, and cell-free supernatants [37,38]. The reaction mixtures were incubated at 37 °C for 90 min. The absorbance was subsequently measured at 317 nm, to determine the extent of radical neutralization. *L. casei* was employed as control strain.

##### Superoxide Radical Scavenging Activity

The superoxide anion radical (O_2_^•−^) scavenging activity was determined in intact cells, cell lysates and cell-free supernatants according to the pyrogallol autoxidation method [37,38]. After the reaction period, the process was terminated, and the absorbance was measured at 325 nm. All experiments were performed in triplicate, utilizing *L. casei* as a control. The scavenging efficiency was calculated based on the inhibition rate of pyrogallol autoxidation, as previously reported [37,38].

#### 2.3.3. Viability of *Lactobacillus* and *Bifidobacterium* from *Gut microbiota*

Bacterial viability was evaluated using the same consortiums described for the antioxidant activity assays. The effect of ESPs was tested at concentrations of 800, 1200, and 1600 µg/mL. *L. casei* and *B. animalis sub lactis* BB12 were employed as reference control strains. Cell viability was quantified via the standard plate count method [37,38]; briefly, aliquots were collected at specific time intervals (0, 4, 6, 8, 12, and 24 h) and inoculated onto MRS agar. Plates were incubated at 37 °C for 48 h under anaerobic conditions. Results are expressed as colony-forming units per milliliter (CFU/mL) to establish the growth kinetics for each treatment.

### 2.4. Statistical Analysis

Normality and homoscedasticity were determined, using Prism-GrapdhPad v.8.0.1, due to the distribution of the data. A Kruskal–Wallis test with *p* ≤ 0.05 was conducted. Significant differences were found by Dunns’ test. Particularly, the one-tailed Mann–Whitney test with *p* ≤ 0.05 was used to analyze significant differences in gene expression between LPS regarding diclofenac + LPS or ESPs + LPS.

## 3. Results

### 3.1. Maturation of C. marginatum

**Figure 1:**
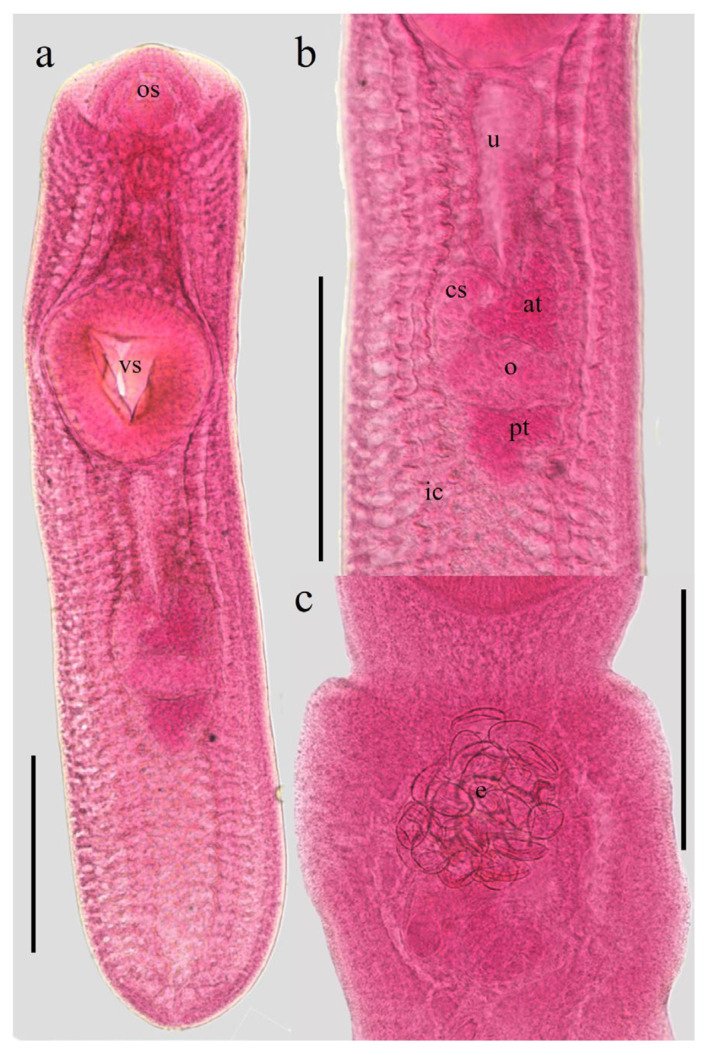
Metacercariae (a life cycle stage of trematodes that represents the infective for the host) of *C. marginatum* isolated from fish *D. maculatus* captured in a freshwater body near Tlacotalpan, Veracruz. (**a**) Body. (**b**) Genital complex. (**c**) Eggs within the uterus after 30 days of in vitro cultured in RPMI-640 medium. Abbreviation: os: oral sucker; vs: ventral sucker; u: uterus; cs: cirrus sac; at: anterior testis; o: ovary; pt: posterior testis; ic: intestinal caeca; e: eggs. Scale bars: 0.5 mm.

At the onset of the in vitro culture, the metacercariae exhibited morphological features characteristic of the species and its respective developmental stages (Figure 1a,b). A survival rate of 74% was maintained over the two-month experimental period. Additionally, the formation of a white precipitate was macroscopically evident in the culture medium, which was subsequently confirmed to contain ESPs, which did not exhibit pathognomonic signs of senescence or death, such as integumentary discoloration or structural morphological degradation. Notably, after 30 days of incubation, an average of 20 eggs were identified within the uterine cavity of several specimens (Figure 1c), suggesting maturation under the established culture conditions.

#### Isolation and Characterization of Helminth-Derived Products

**Figure 2:**
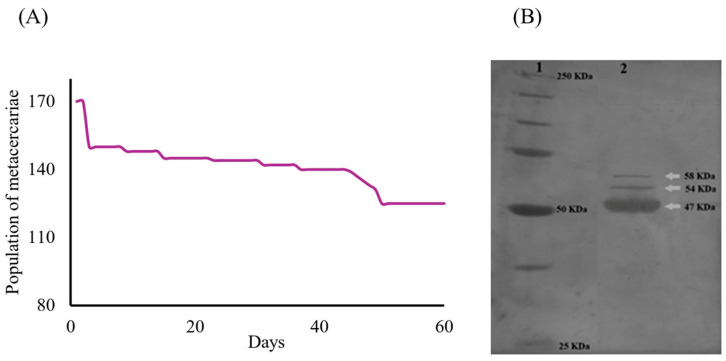
Survival and electrophoretic characterization of proteins from *C. marginatum* metacercariae ESPs. (**A**) Survival curve during the 60-day in vitro experiment (*ni* = 170 and *nf* = 126). (**B**) Separation profile of proteins from ESPs using 10% SDS-PAGE of a sample of ESPs from eight cuts of *C. marginatum* metacercariae cultures. Line 1 shows the molecular weight markers ranging from 25 to 250 kDa. Line 2 shows the electrophoretic pattern (separation and characterization) of the mixed ESPs collected from the eight cuts. Protein bands with apparent molecular weights of 47, 54, and 58 kDa are observed.

The survival rate of the metacercariae population (*n_i_* = 170) throughout the trial was recorded at 74% (*n_f_* = 126). Two distinct decrements in survival were observed on days 3 and 49. The first decline at the onset of the trial was likely associated with mechanical stress during medium replacement. The second decline was probably attributed to the acidification of the culture medium. Nevertheless, the cumulative mortality rate was limited to 26% (*n* = 44) (Figure 2A). Analysis of the ESPs identified from the *C. marginatum* culture supernatants collected over a 60-day period revealed three prominent protein bands (Figure 2B). These proteins exhibited estimated molecular weights of 47, 54, and 58 kDa. This protein profile remained consistent across the eight collection intervals throughout the 60-day culture period, with an average protein concentration of 104.01 mg/mL. A total volume of 60 mL of ESP-enriched medium was harvested by the end of the experiment.

### 3.2. In Vitro Evaluation of the Biological Effects of ESPs

**Figure 3:**
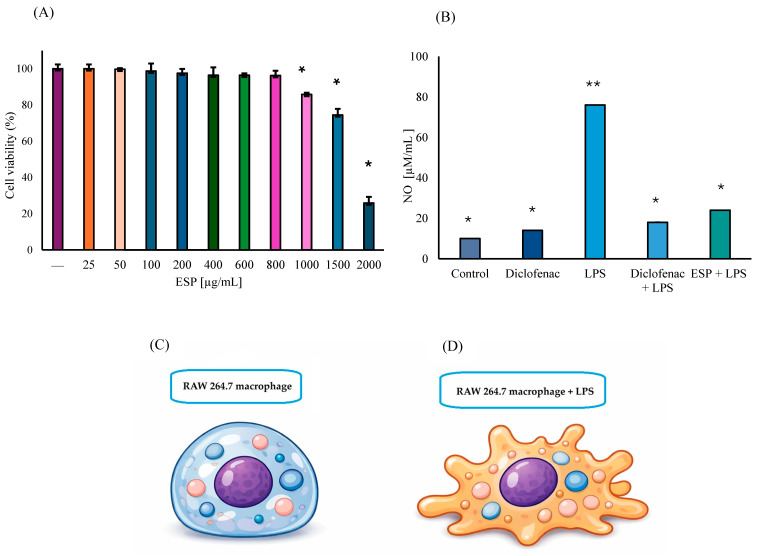
Biological activity of ESPs (**A**) Cell viability of RAW 264.7 MΦ treated with ESPs (25–2000 μg/mL). Control cells represent 100% viability. Data represent three independent experiments and are expressed as the means ± SD. Asterisk (*) denotes significant differences (*p* ≤ 0.01) relative to the control. (**B**) Quantification of NO production in the anti-inflammatory assay using MΦ supernatants and 800 µg/mL of ESPs. Asterisk versus double asterisk (* vs. **) denotes significant differences relative to LPS. (**C**) Schematic representation of RAW 264.7 macrophages in basal state with rounded morphology. (**D**) Macrophage stimulated with LPS, showing extension of pseudopods, and increased cellular irregularity.

The viability of RAW 264.7 murine MΦ treated with ESPs at concentrations ranging from 25 µg/mL to 800 µg/mL showed no significant reduction compared to untreated control cells. A minor decrease of 14% was observed at 1000 µg/mL. However, cell viability was significantly compromised at concentrations of 1500 and 2000 µg/mL, diminishing by 26% and 74%, respectively (Figure 3A). Regarding NO production, the control group exhibited minimal levels, representing the basal metabolic state of unstimulated MΦ. Interestingly, cells exposed to diclofenac alone showed a slight 18% increase in NO production (*p* ≤ 0.01), compared to the control group. As expected, LPS stimulation induced an 87% increase in NO levels (*p* ≤ 0.01), which was considered the 100% reference for the induced inflammatory response. Furthermore, pronounced morphological changes, specifically the formation of pseudopods, were evident in cells exposed to only LPS (ii group) (Figure 3C,D).

In the group of diclofenac + LPS, NO production decreased notably by 76% relative to the LPS-only group, which represents a 5% reduction when compared to the diclofenac basal group (*p* ≤ 0.01). These results validated the sensitivity of the experimental model to anti-inflammatory modulation. Most importantly, NO production was significantly suppressed by 68% (*p* ≤ 0.01), in the ESPs + LPS group compared to the LPS group, which corresponds to a 5.2-fold decrease in NO levels, strongly suggesting an anti-inflammatory effect (Figure 3B). It should be noted that the ESP concentration within the range of 50 to 600 µg/mL did not exhibit significant anti-inflammatory activity.

#### Pro-Inflammatory Gene Expression Levels

**Figure 4:**
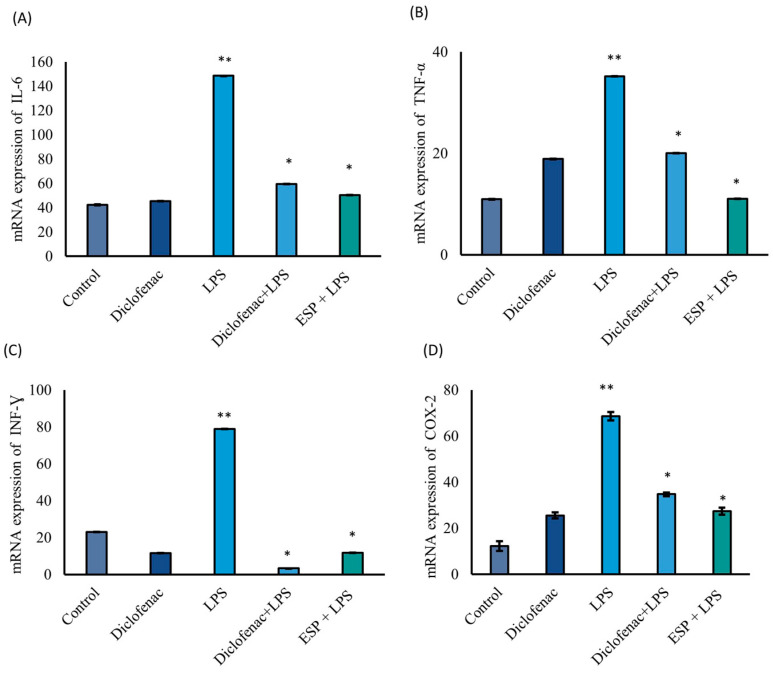
mRNA expression levels of pro-inflammatory cytokines in RAW 264.7 MΦ. (**A**) IL-6; (**B**) TNF-α; (**C**) INF-γ; (**D**) COX-2. Asterisk vs double asterisks (* vs. **) denote significant differences between the LPS group and all other treatments (*p ≤* 0.05) for each gene expression assay. Target genes were normalized to 18S mRNA. Data represent three independent experiments and are expressed as the mean ± SD.

The mRNA expression levels of IL-6, TNF-α, INF-γ, and COX-2 were significantly downregulated by 66%, 69%, 97%, and 60%, respectively, compared to the LPS group, which served as the 100% inflammatory response (Figure 4A–D). Notably, in the ESPs + LPS group, ESP treatment yielded a reduction in IL-6 (66%) and TNF-α (69%) expression that was comparable to, or greater than, the effects observed in the diclofenac + LPS group (60% and 43% reduction, respectively). Regarding IFN-γ (Figure 4C), its expression was suppressed by 85% in the ESPs + LPS group relative to the LPS-stimulated group; remarkably, this level was 48% lower than the expression observed in the untreated control (basal) group. Furthermore, COX-2 mRNA levels were significantly reduced by 60% in the ESPs + LPS group compared to the LPS group. This inhibitory effect on the COX-2 mediator was similar in magnitude to the diclofenac group (Figure 4D). Interestingly, the ESPs + LPS group exhibited a more pronounced suppression of COX-2 expression (only 39% residual expression) compared to the diclofenac + LPS group (51%).

### 3.3. Antioxidant Effect of the Consortium of Lactobacillus and Bifidobacterium with ESPs

**Figure 5:**
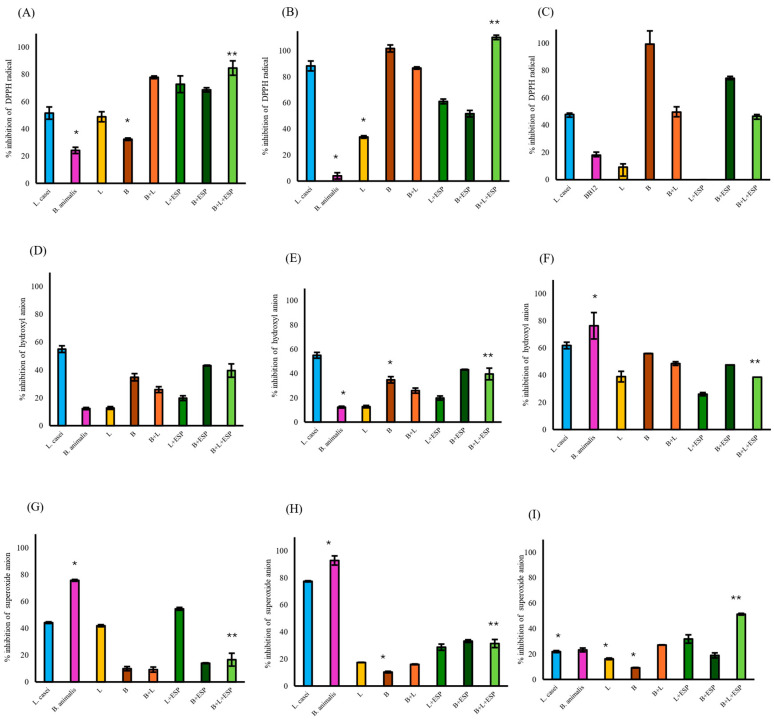
Antioxidant activity of different cellular fractions from gut bacteria supplemented with 800 µg/mL ESPs. *L. casei* and *B. animalis* BB12 were employed as reference control strains. L: consortium of *Lactobacillus* (1 × 10^8^ CFU/mL); B: consortium of *Bifidobacterium* (1 × 10^8^ CFU/mL). (**A**) DPPH radical scavenging in intact cells. (**B**) DPPH radical scavenging in cell-free supernatant. (**C**) DPPH radical scavenging in cell lysates. (**D**) Hydroxyl radical scavenging in intact cells. (**E**) Hydroxyl radical scavenging in cell-free supernatant. (**F**) Hydroxyl radical scavenging in cell lysates. (**G**) Superoxide radical scavenging in intact cells. (**H**) Superoxide radical scavenging in cell-free supernatant. (**I**) Superoxide radical scavenging in cell lysates. Data represent three independent experiments and are expressed as the mean ± SD. Significant differences are indicated from the B + L consortium + ESPs group relative to all other experimental groups. An asterisk (*) or double asterisk (**) denote statistical significance at *p ≤* 0.05.

Among the cellular fractions evaluated, the supernatant of the B + L + ESPs group exhibited the highest DPPH radical scavenging activity (110%, Figure 5B), followed by intact cells (85%, Figure 5A). In contrast, the activity of cell lysates in the same group was recorded at 47% (Figure 5C). Regarding the hydroxyl radical, the B + L + ESPs group showed scavenging activities of 40% in intact cells, 31% in cell-free supernatants, and 39% in lysates. However, consistently higher hydroxyl scavenging activity was observed in the B + ESPs group in intact and cell lysates (Figure 5D–F).

Superoxide anion scavenging in the B + L + ESPs group (17%) was significantly lower (*p* ≤ 0.05) compared to the L + ESPs group (55%) in intact cells (Figure 5G). In the cell-free supernatant, the B + L consortium +ESPs showed significantly higher inhibition (31%, *p* ≤ 0.05) than the L + ESPs consortium (29%, Figure 5H). The maximum superoxide scavenging activity was identified in the cell lysates of the B + L consortium + ESPs (51%, Figure 5I).

When the B + L + ESPs group was compared with the control groups (*L. casei* and *B. animalis* BB12), significant differences (*p* ≤ 0.05) were observed with respect to *B. animalis* BB12 in all fractions except DPPH and superoxide radicals in lysed cells (Figure 5C,I) and hydroxyl radicals in intact cells (Figure 5D). *L. casei* showed significant differences (*p* ≤ 0.05) in superoxide radicals in cell lysates (Figure 5I).

#### Viability of Bacteria from Bacterial Consortiums Regarding ESPs

**Figure 6:**
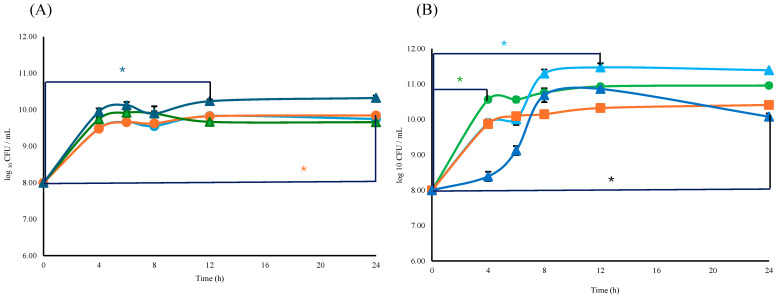
Effect of ESPs over the viability of *Lactobacillus* and *Bifidobacterium*. (**A**) L and B consortium with and without ESPs: 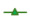 B consortium, 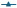 B consortium + ESPs 800 µg/mL, 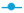 L consortium, and 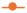 L consortium + ESPs 800 µg/mL. * Dark blue and * orange asterisks means significant difference (*p* ≤ 0.05) between B + ESM 800 μg/mL/0 h vs. B + ESM 800 μg/mL/12 h and between L + ESM 800 μg/mL/0 h vs. L + ESM 800 μg/mL/24 h. (**B**) Viability of Bifidobacterium + Lactobacillus with ESPs 800 µg/mL, 1200 µg/mL, and 1600 µg/mL: 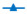 B + L consortium, 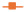 B + L consortium + ESPs 800 µg/mL, 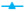 B + L consortium + ESPs 1200 µg/mL, 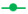 B + L consortium + ESPs 1600 µg/mL. * Black, * green, and * blue asterisks indicate significant differences (*p* ≤ 0.05) between B + L/0 h vs. B + L + ESM 800 µg/mL/24 h; B + L/0 h vs. B + L + ESM 1600 µg/mL/4 h and B + L/0 h vs. B + L + ESM 1200 µg/mL/12 h respectively. Data represent three independent experiments and are expressed as the mean ± SD.

The viability of either *Lactobacillus* or *Bifidobacterium* consortium with respect to the L consortium or B consortium + ESPs was similar (*p ≥* 0.05). The specific growth rate (*μ* = h^−1^) and doubling time (*t_d_*) for the *Bifidobacterium* consortium were *μ*_B_ = 1.09 h^−1^ and *t_d_*_B_ = 0.63 h, respectively; for *Lactobacillus, μ*_L_ and *t_d_*_L_ were 1.12 h^−1^, and 0.62 h respectively. The supplementation with 800 µg/mL of ESPs resulted in a specific growth rate to *Bifidobacterium of μ*_B+ESPs_ = 1.20 h^−1^ and doubling time of *t_d_*_B+ESPs_ = 0.62 h. In the case of *Lactobacillus*, they were *μ*_L+ESPs_ = 1.13 h^−1^ and *t_d_*_L+ESPs_ = 0.61 h (Figure 6A). Significant differences (*p* ≤ 0.05) were observed in B consortium + ESPs (800 µg/mL) between times of 0 and 12 h. In the case of L consortium, significant differences were observed at the same ESP concentration but between times of 0 and 24 h (Figure 6A).

For the B + L consortium, the specific growth rate was *μ*_B+L_ = 1.24 h^−1^ with a doubling time of *td*_B+L_ = 0.56 h. When supplemented with 800 µg/mL of ESPs, the consortium exhibited a specific growth rate of *μ*_B+L+ESPs_ = 1.18 h^−1^ and a doubling time of *t_d_*_B+L+ESPs_ = 0.59 h. The presence of ESPs to 1200 µg/mL (*μ*_B+L_ = 1.24 h^−1^, *td*_B+L_ = 0.56 h) did not affect the specific growth rate compared to the B + L consortium. When 1600 µg/mL of ESPs was used, the specific growth rate decreased (*μ*_B+L_ = 1.18 h^−1^, *td*_B+L_ = 0.59) compared to the B + L consortium, while a significant difference in viability was found at 4 h (*p* ≤ 0.05) (Figure 6B). Significant differences in cell viability were observed between the B + L consortium and the ESP-supplemented groups as a function of increasing concentration. Supplementation with 1200 µg/mL ESPs resulted in significant variations between 0 and 12 h, while the 1600 µg/mL concentration showed significant differences within the first 4 h of incubation (*p* ≤ 0.05) (Figure 6B). These findings suggest a dose-dependent effect of ESPs on the early stages of the consortium’s growth kinetics.

## 4. Discussion

The in vitro cultivation of helminths, especially trematodes, remains technically challenging due to the complexity of reproducing the host-dependent physiological cues required to sustain their development and complete reproductive cycle. In this work, RPMI-1640 medium supported the development of approximately 20 intrauterine eggs after 30 days, concomitant with ESP production. This indicates partial reproductive activity in a system that still insufficiently mimics host-dependent signaling. RPMI-1640 has been widely used to sustain helminth viability and maturation of larval to egg-producing stages [26,40]. Although metacercariae can be maintained under laboratory conditions, egg production is typically constrained, and eggs are frequently malformed, as reported for species such as *Echinostoma caproni* and other digeneans [41,42]. This developmental limitation is largely attributed to the absence of host-specific stimuli, such as transforming growth factor-beta (TGF-β) and other signaling molecules essential for triggering full reproductive functionality, which are characteristically lacking in conventional culture media [43,44].

The three proteins identified in ESPs (Figure 2B) fall within the molecular weight range reported for secreted proteins from other helminth species [45], and it is possible that these proteins exhibit distinct biological activities. However, the biological effects observed in this study must be attributed to the total mixture rather than exclusively to the specific proteins identified, which require further research. Excretory-secretory products constitute a complex mixture of bioactive molecules wherein combined or synergistic interactions may orchestrate the observed effects.

Regarding cell viability, macrophages were not affected by the ESPs, at concentrations below 800 µg/mL. A reduction in macrophage viability was observed at concentrations higher than 1000 µg/mL, suggesting cytotoxicity at supra-physiological doses; however, the mechanism underlying this effect in *C. marginatum* ESPs remains undefined and requires targeted evaluation.

ESPs released by helminths exhibit diverse biological properties, including anti-inflammatory effects [20], with activities that, in some cases, are comparable to those of pharmacological agents while offering a potentially safer profile [20,41]. A recognized mechanism underlying these effects involves the modulation of inflammatory pathways through the regulation of iNOS expression and the consequent control of iNOS-derived nitric oxide, leading to the attenuation of NO-dependent inflammatory responses [20]. LPS, a potent pro-inflammatory agonist, drives macrophage activation by inducing iNOS expression and sustained NO production; thus, RAW 264.7 cells constitute a robust system for evaluating immunoregulatory properties. In the present study, NO secretion in LPS-stimulated RAW 26.7 macrophages were reduced by 76% following exposure to *C. marginatum* ESPs. This significant inhibition suggests a modulatory effect on macrophage activation under these specific experimental conditions, highlighting the possible anti-inflammatory potential of the helminth-derived products. Gadahi et al. (2016) [46] reported the suppression of NO and INF-γ production, along with an increase in anti-inflammatory cytokines, mediated by a 24 kDa HcESP protein secreted by *Haemonchus*. Following the NO suppression observed in RAW 264.7 cells, comparable anti-inflammatory effects have been linked to ESPs from other helminths within similar molecular weight ranges. For example, 45, 49, and 53 kDa ESPs from *Trichinella spiralis* inhibited pro-inflammatory cytokines and increased IL-4, IL-10 and TGF-β1 in independent macrophage systems, where mediator modulation was associated with a shift from M1-toward M2-like profiles [45,47]. Additionally, *Steinernema carpocapsae* and *Heterorhabditis bacteriophora*, which produce ESPs of 25–83 kDa, have been contributed to a significant NO reduction in LPS-activated macrophages (>70%) [25,48]. In this study, diclofenac was included as an anti-inflammatory control due to its well-established inhibitory effect on COX-2 and its downstream regulation of iNOS-mediated NO synthesis. Consistent with this expected response, a significant reduction in extracellular NO levels was observed in LPS-stimulated RAW 264.7 macrophages (Figure 3B), confirming the suitability and sensitivity of the inflammatory in vitro model. Like the findings reported for *E. granulosus* ESPs [48], our study demonstrated a significant reduction in NO secretion following exposure to *C. marginatum* ESPs. In this context, Mendoza-Rodríguez et al. (2024) [49] reported that the ESPs of *Taenia crassiceps* reduced NO levels by more than 70%, a finding consistent with the results obtained in our study. These authors also observed a significant decrease in pro-inflammatory cytokines in LPS-stimulated macrophages, which correlates with our findings of reduced mRNA expression for IL-6, TNF- α, INF-γ, and COX-2. Similarly, Yang et al. (2024) [35] demonstrated that recombinant stefins from the helminth *Cysticercus pisiformis* exhibited anti-inflammatory properties by suppressing the gene expression of IL-1, IL-6, TNF-α, and COX-2 in LPS-induced RAW264.7 macrophages.

Collectively, it has been shown that inflammatory microenvironments are characterized by elevated INF-γ levels, which promote the overproduction of TNF-α and other pro-inflammatory cytokines [50]. Consistent with previous reports, LPS stimulation markedly increased INF-γ and TNF-α gene expression, confirming the induction of a pro-inflammatory state in RAW 264.7 cells [50,51]. Conversely, exposure to *C. marginatum* ESPs significantly attenuated the expression of these mediators (Figure 4B,C), reinforcing their ability to ESPs to modulate canonical inflammatory pathways. ESPs have shown capacity to modulate cellular redox responses, attenuating excessive production of ROS and inflammatory mediators in macrophage models [52]. The interaction between helminth-derived secretions and host or microbial antioxidant systems is an emerging area of interest, as redox imbalance plays a key regulatory role in macrophage inflammatory activation and its subsequent resolution.

In the present study, the integration of intact cells from the *Bifidobacterium* consortium with ESPs resulted in a significant enhancement (*p* ≤ 0.05) of DPPH radical scavenging activity compared to the baseline levels. This augmented antioxidant response suggests that the presence of ESPs could potentiate the free-radical neutralizing capacity of the probiotic consortium, possibly through surface or membrane-associated mechanism.

A similar trend was observed in the cell-free supernatant fraction (*p* ≤ 0.05); however, this enhancement was specifically evident for the *Lactobacillus* consortium, suggesting that the metabolic or secretory interactions between ESPs and probiotics may be genus-specific. The same effect was observed in cell-free supernatants but in the *Lactobacillus* consortium. *Lactobacillus* strains can exhibit DPPH scavenging activity through various mechanisms, one of them through the release of antioxidant metabolites, such as exopolysaccharides, peptides, or organic acids; another by surface associated antioxidant enzymes, including superoxide dismutase or catalase-like activities; and through cell wall components, particularly peptidoglycan and teichoic acids, which may act as electron donors [53,54]. In the case of exopolysaccharides, they modulate cellular antioxidant signaling pathways which are related to the success of host colonization by parasites [55], supporting their functional role. The hydroxyl radical is one of the most reactive and harmful free radicals in biological systems; it can cause severe oxidative damage to nucleic acids, lipids, and proteins due to its high reactivity and ability to initiate chain reactions [56]. In the present study, intact cells exhibited limited antioxidant inhibition, likely due to structural cellular barriers that sequester intracellular antioxidants, thereby restricting their radical-scavenging accessibility. In contrast, the cell-free supernatant demonstrated superior hydroxyl radical scavenging activity, with a notable improvement observed in the *Bifidobacterium* consortium upon ESP supplementation. This suggests that water-soluble metabolites with electron-donating capacity are primarily responsible for the observed effects, which is consistent with previous studies highlighting the antioxidant role of *Lactobacillus*-derived exopolysaccharides, bioactive peptides, and phenolic acids [54,57,58]. This pattern supports a synergy between extracellular fractions of probiotic bacteria and *C. marginagtum* ESPs, suggesting promising applications to manage antioxidants [59].

Probiotic strains, particularly from the *Lactobacillus* genus, scavenge hydroxyl radicals through diverse mechanisms, including the production of antioxidant metabolites such as glutathione, organic acids, and exopolysaccharides as well as metal ion chelation and enzymatic activities, including NADH oxidase or peroxidase-like actions [60,61,62]. Furthermore, cell surface components may act directly as radical scavengers [63]. The superoxide anion is an ROS generated by enzymes such as NADPH oxidases in intestinal epithelial and immune cells. An overproduction of O^2−^ contributes to redox imbalance and may exacerbate inflammatory processes [64]. Consequently, the modulation of O^2−^ by gut microbiota components represents a significant area for understanding intestinal redox homeostasis [65]. Intact *Lactobacillus* consortium cells exhibited strong superoxide anion inhibition, aligning with studies reporting constitutive membrane- or cell surface- associated SOD activity in *Lactobacillus* species under non-lysed conditions [62,63,66]. In this study, the highest significant *(p ≤* 0.05) superoxide scavenging inhibition was in the group combining *Lactobacillus* and ESPs in lysed cells, consistent with the interaction between ESPs and bacterial intracellular antioxidant signaling pathways, potentially involving in Nrf2 and SOD [67,68,69].

The observation that the B consortium showed enhanced growth when supplemented with ESPs at 24 h suggests that the ESPs act as a trophic factor or source of nutrients for the *Bifidobacterium* genus [68]. These findings align with literature reporting that *Bifidobacterium* species can utilize a wide variety of compounds, including glycoconjugates and peptides, for accelerated growth under culture conditions. While complex in vivo effects, such as the promotion of mucin expression, or epithelial protection were not evaluated in this in vitro model, the ability of the ESPs to improve bacterial viability in the culture medium represents a key support mechanism [70]. In contrast, *Lactobacillus* spp. are often more adapted to mildly acidic, dynamic, and even proinflammatory environments. In fact, many *Lactobacillus* strains rely on competitive exclusion mechanisms that depend on the production of lactic acid, bacteriocins, and hydrogen peroxide [71]. Consequently, the enhanced trophic effect observed with ESPs in the B consortium was not replicated with L consortium, suggesting that growth potentiation provided by the ESPs is not the primary mechanism for the survival or competitive success of these *Lactobacillus* strains. Recent proteomic studies have identified helminth ESPs that inhibit antimicrobial peptides (AMPs) like RegIIIγ and defensins; this selective suppression of host antimicrobials creates a niche in which bifidobacteria are better suited to thrive than lactobacilli [72,73]. Co-culturing B + L consortium with ESPs showed dose-dependent growth. These results suggest that while ESPs are biocompatible during the active growth phase, higher concentrations may accelerate the transition toward the stationary phase or limit long-term cell survival. These findings align with research indicating that helminth ESPs contain microbial peptides and immunomodulatory immune-modulatory factors capable of reshaping the gut microbiota [16,74,75]. In vitro studies have demonstrated that helminth ESPs can exert direct effects on bacterial growth that vary by genus.

An experiment with ESPs of *Nippostrongylus brasiliensis* adults showed broad-spectrum bactericidal activity against Gram-positive and negative species, in a dose-dependent and heart-resistant manner, indicative of protein-mediated antibacterial factors [76,77]. Interestingly, under controlled in vitro conditions, certain anaerobic commensals such as *Bifidobacterium* spp. exhibit enhanced tolerance and may even proliferate when exposed to sub-lethal concentrations of ESPs. This response is plausibly attributed to intrinsic resistance mechanisms or protective metabolic pathways capable of degrading helminth-derived proteins. Conversely, *Lactobacillus* spp. displayed reduced resilience, potentially due to heightened sensitivity to specific ESP components, including protease inhibitors or glycoproteins, which may compromise cell wall integrity or interfere with essential metabolic pathways [77]. Additionally, in vitro studies using larval ESPs from *Echinococcus multilocularis* have shown that these molecules can induce apoptosis or functional inhibition in immune-like cells, supporting the feasibility of direct molecular interactions between helminth ESPs and microbial receptor analogs including bacterial surface structures [78]. Collectively, these findings revealed that helminth ESPs contain protein-based factors capable of selectively modulating microbial viability or growth. A complementary perspective emerges from in vitro probiotic-helminth interaction *models*, in which probiotic mixtures containing *Bifidobacterium* spp. become enriched upon helminth exposure, whereas *Lactobacillus* responses vary according to both mixture composition and helminth species (e.g., *Oesophagostomum dentatum* with pig probiotics, where effects were more pronounced in *Bifidobacterium*) [75,79]. These observations suggest a potential functional compatibility between bifidobacterial metabolism and helminth ESP components, possibly mediated by proteolytic capacity or superior stress tolerance—traits that may be comparatively less efficient or absent in *Lactobacillus* spp.

Helminth ESPs include glycoproteins and enzymes capable of reshaping the gut microbiota, frequently promoting the enrichment of beneficial bacterial groups [80]. Certain ESP molecules have also been shown to interact directly with bacterial receptor analogs, influencing pathways related to energy metabolism, stress tolerance, and biofilm dynamics [81]. To fully elucidate the biological role of ESPs in *C. marginatum*, future research should profile bacterial and host-derived metabolites, immune gene expression, and whole-microbiome community shifts. These effects must ultimately be validated in vivo using robust animal or clinical models. A comprehensive understanding of ESP function in *C. marginatum* will require the incorporation of multiple complementary lines of evidence. Subsequent studies should include characterization of the fecal microbiota of the definitive host species to determine whether the detected proteins derive exclusively from helminth metabolism or are partially contributed by the helminth-associated microbiota, acknowledging that the helminths harbor complex endogenous microbial communities for which they serve as ecological hosts. High-resolution proteomic profiling of ESPs will be essential to define their molecular composition and identify putative bioactive factors. Parallel taxonomic and functional analyses of the helminth-associated microbiota will further clarify the microbial contribution to the overall ESPS protein repertoire. Evaluating the immunomodulatory potential of ESPs within *Lactobacillus* culture systems provides preliminary evidence of their biological activity. Our observations in RAW 264.7 macrophages suggest a possible modulatory effect on host immune-associated signaling; however, further in vivo studies are required to confirm a definitive anti-inflammatory role. These initial findings could indicate that ESP components selectively interact with beneficial gut bacteria while concurrently influencing host immune responses, though the full extent of this therapeutic potential remains to be elucidated.

The absence of endotoxin quantification and protein inactivation controls represents a limitation of the present study. Consequently, the biological effects reported herein should be interpreted as being associated with the biochemical complexity of the ESP mixture rather than attributed to discrete, individual helminth products. This perspective acknowledges the potential contribution of non-proteinaceous components or synergistic interactions. In this framework, ongoing work involves global proteomic identification and functional annotation of the proteins within the ESPs, together with in-depth characterization of the parasite-associated microbiota. These complementary analyses will be presented in a subsequent publication.

## 5. Conclusions

This study identified three proteins within the ESPs of *C. marginatum* exhibiting dual biofunctional properties: a potent anti-inflammatory activity and the ability to enhance the viability and antioxidant capacity of defined probiotic consortia under in vitro conditions. The ESPs significantly reduced nitric oxide production and downregulated inflammatory mediators. Furthermore, the ESPs increased free-radical scavenging activity in both extracellular and intracellular bacterial fractions and promoted bacterial viability, particularly in *Bifidobacterium*-based consortia, while responses in *Lactobacillus* were strain-and dose-dependent. These effects were evaluated using standardized initial bacterial populations and should be interpreted as functional responses at the consortium level. Overall, helminth-derived products represent a promising source of multifunctional bioactive compounds; our findings provide a preliminary basis for understanding the biochemical interaction between helminth-derived products and gut microbiota. Further in vitro and mechanistic studies are required to elucidate the molecular pathways involved in ESP-bacteria-host interactions and to support their potential role as bioactive modulators.

## Figures and Tables

**Table 1:** Sequence of nucleotides of the specific primers for qRT-PCR.

Gene	Forward	Reverse	Length bp	Reference
IL-6	5-GAGGATACCACTCCCAACAGAC-3	5-AAGTGCATCATCGTTGTTCATACA-3	129	[32]
INF-γ	5-GCTCTGAGACAATGAACGCT-3	5-AAAGAGATAATCTGGCTCTGC-3	227	[33]
TNF-α	5-CCCTCACACTCACAAACCACCA-3	5-TGAGGAGCACGTAGTCGGGG-3	154	[34]
COX-2	5-TGTGACTGTACCCGGACTGG-3	5-TGCACATTGTAAGTAGGTGGAC-3	233	[35]
18S	5-CGGACACGGACAGGATTGACA-3	5-CCAGACAAATCGCTCCACCAACTA-3	94	[36]

## Data Availability

The original contributions presented in the study are included in the article. Further inquiries can be directed to the corresponding authors.

## Abbreviations

The following abbreviations are used in this manuscript:

AMPs	Antimicrobial peptides
cs	Cirrus sac
CDDs	Chronic degenerative diseases
CH_2_O	Formaldehyde
CH_3_COOH	Acetic acid
(CH_3_)_2_SO	Dimethyl sulfoxide
CO_2_	Carbon dioxide
COX-2	Cyclooxygenase-2
DNA	Desoxyribonucleic acid
DPPH	2,2-diphenyl-1-picrylhydrazyl radical
Dt	Duplication time
e	eggs
ESPs	Excretory secretory proteins
HCl	Hydrochloric acid
ic	Intestinal caeca
IL-6	Interleukin-6
IL-10	Interleukin-10
IL-12	Interleukin-12
IL-23	Interleukin-23
IL-1β	Interleukins-1β
INF-γ	Interferon gamma
iNOS	Nitric oxide synthase
LAB	Lactic acid bacteria
LPS	Lipopolysaccharide
Na_2_CO_3_	Sodium carbonate
NADH	Nicotinamide adenine dinucleotide
NADPH	Nicotinamide Adenine Dinucleotide Phosphate
ni	Initial survival rate of the metacercariae population throughout the trial
nf	Final survival rate of the metacercariae population throughout the trial
NF-κB	Nuclear factor enhancing the kappa light chains of activated B cells
NO	Nitric oxide
Nrf2	Erythroid-derived nuclear factor 2
NSAIDs	Nonsteroidal anti-inflammatory drugs
μ	Specific growth rate
MAPK	Mitogen-activated protein kinases
MΦ	Macrophages
MRS	De Man Rogosa Sharpe medium
MTT	3-(4,5-dimethyl-2- thiazolyl)-2,5-diphenyl-2H-tetrazolium bromide
o	ovary
OD	Optical density
os	Oral sucker
⸱OH	Hydroxyl radical
O^2−^	Superoxide anion radical
PAGE	Polyacrylamide gel electrophoresis
PBS	Phosphate-buffered saline
PGs	Producing prostaglandins
pt	Posterior testis
qRT-PCR	quantitative real-time
RegIIIγ	Regenerating islet-derived protein III-gamma
RNA	Ribonucleic acid
ROS	Reactive oxygen species
RPMI-1640	Roswell Park Memorial Institute 1640 medium
SD	Standard deviation
SOD	Superoxide dismutase
TGF-β	Transforming growth factor beta
Th1	T helper type 1 cells
Th17	T helper type 17 cells
TNF-α	Tumor necrosis factor alpha
u	Uterus
vs	Ventral sucker
^ΔΔ^Ct	Delta delta Ct
